# Spatiotemporal complementarity synergistically drives high productivity in intercropping systems: a comparison of grain maize and fresh maize intercropped with soybean

**DOI:** 10.3389/fpls.2026.1932793

**Published:** 2026-10-12

**Authors:** Jie Li, Meng Kong, Dongmei Zhang, Mingjing Huang, Kexing Hao

**Affiliations:** 1 Shanxi Institute of Organic Dryland Farming, Shanxi Agricultural University/Shanxi Provincial Key Laboratory of Organic Dryland Agriculture, Taiyuan, China; 2 School of Agricultural Economics and Management, Shanxi Agricultural University, Taiyuan, China

**Keywords:** complementarity effect, economic benefit, intercropping, interspecific competition, land equivalent ratio, net effect, selection effect

## Abstract

Intercropping is an important planting pattern for improving land use efficiency. However, current research and practical applications focus mainly on the performance of the main crop and overall system yield, while neglecting the growth constraints on companion crops, which are often weaker competitors. This oversight contributes to critical challenges in intercropping systems, including imbalanced interspecific competition and substantial yield losses in companion crops. We conducted a three-year field experiment in the semi-arid rainfed agricultural region of northern China. We evaluated the productivity (crop yield, land equivalent ratio [LER], net effect), interspecific competition characteristics (competitive ratio, aggressivity), and economic benefits of the two intercropping systems: grain maize/soybean (GM/S) and fresh maize/soybean (FM/S). A central objective was to elucidate yield compensation response of subordinate soybean and the regulatory mechanism underlying balanced interspecific competition. Our results demonstrated that, averaged over three years, compared with theoretical sole cropping yields, intercropping increased grain maize and fresh maize yields by 25.97% and 17.29%, respectively, but reduced soybean yield. The FM/S system outperformed GM/S; under their optimal configurations, FM/S achieved 3.36% higher LER, and 11.42% higher economic benefits. The main crop was competitively dominant in both systems, but with different intensities: grain maize in GM/S exerted strong competition against soybean (mean CR = 2.07, A = 0.62), whereas fresh maize in FM/S showed milder competition (mean CR = 1.43, A = 0.34). By optimizing spatial configuration and shortening the co-growth period, FM/S improved yield compensation response of subordinate soybean, reducing its mean yield loss to 16.07%—significantly lower than the 37.32% loss in GM/S. Our results suggest that the FM/S intercropping system improves the yield compensation response of subordinate soybean, while enhancing system level complementarity effect (CE). Our observations highlight the combined roles of spatial configuration optimization and temporal niche differentiation, which show distinct advantages over conventional patterns that rely merely on the selection effect. These findings provide an important scientific basis for the optimization, upgrading, and large-scale application of cereal–legume intercropping systems in northern China. We recommend that fresh maize-soybean intercropping be prioritized for promotion in semi-arid rainfed agricultural regions.

## Graphical Abstract

**Figure:**
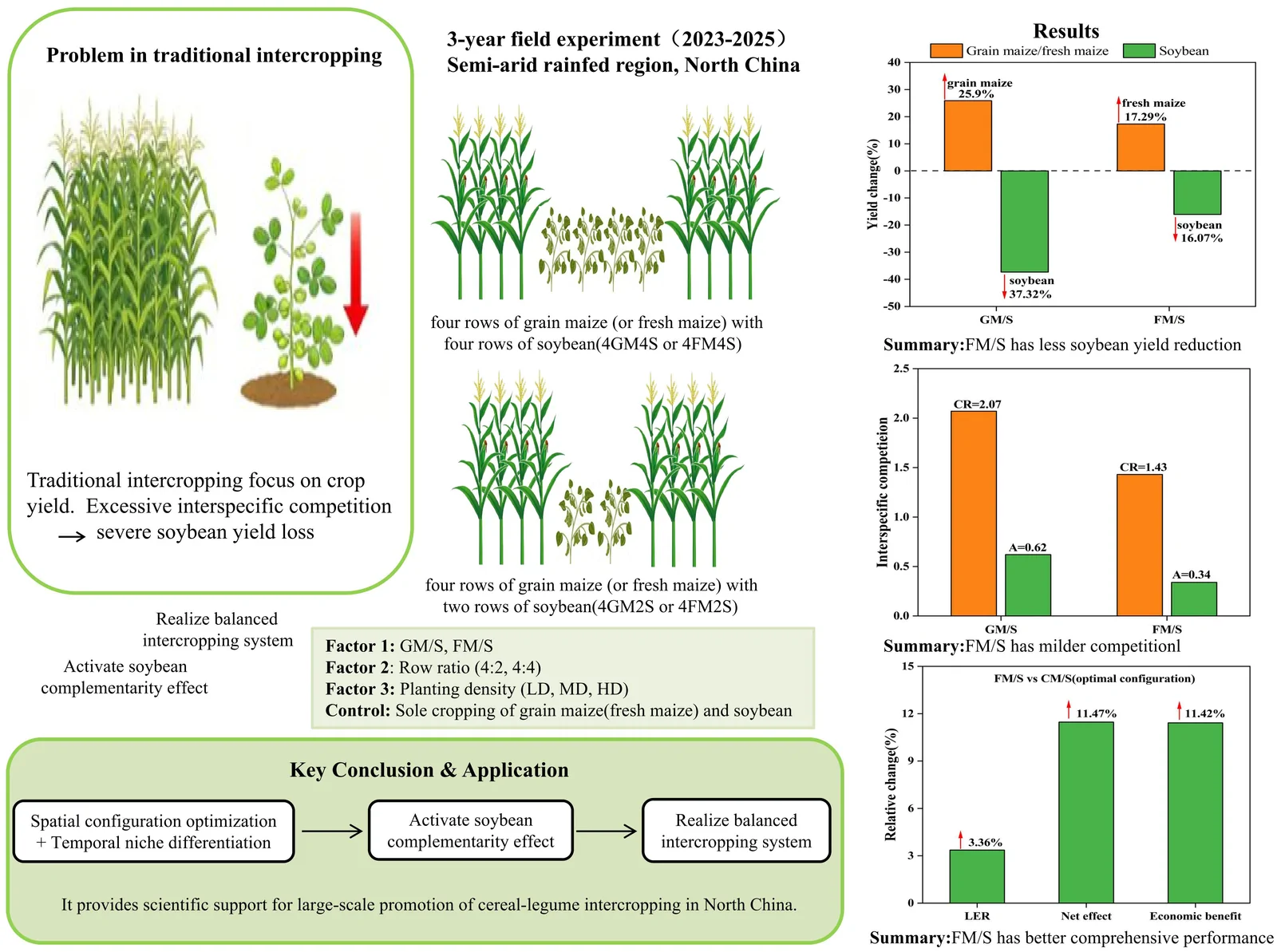

## 1 Introduction

Intercropping is a traditional, intensive agroecological strategy with remarkable productivity and ecological advantages. It improves land-use efficiency (Li et al., 2020b) and optimizes the synergistic utilization of key resources such as light, temperature, water, and nutrients (Liu et al., 2017a; Xu et al., 2020; Qiang et al., 2024; Zhang et al., 2025). Moreover, through interspecific ecological regulation, intercropping suppresses weed proliferation (Gu et al., 2022), mitigates pest and disease damage (Boudreau, 2013), improves soil physicochemical properties (Wang et al., 2025), and reduces synthetic fertilizer and pesticide inputs, thereby synergistically improving both agronomic productivity and ecological benefits (Yan et al., 2024). The yield advantage of intercropping systems stems from the dynamic balance between interspecific complementarity and interspecific competition. This core ecological mechanism can be rigorously quantified and interpreted through the selection effect and the complementarity effect (Loreau and Hector, 2001). The selection effect mainly reflects the yield dominance of highly competitive species, whereas the complementarity effect originates from niche partitioning including both spatial and temporal dimensions. Interspecific competition underpins the selection effect. During the co-growth period, when different crop species compete for limited resources including light, temperature, water, and nutrients, highly competitive dominant crops (e.g., maize) occupy more ecological niches and resources, thereby achieving substantially higher yields that dominate the system’s total productivity. This contribution of competitively superior species constitutes the selection effect. In contrast, shorter-statured subordinate crops (e.g., soybean) exhibit growth suppression due to resource constraints (Li et al., 2020b). Interspecific complementarity is the key prerequisite for the complementarity effect. Intercropped species improve the efficiency of resource utilization through spatiotemporal niche differentiation, including variations in plant height, growth duration, root distribution, and nutrient uptake strategies (Li et al., 2021a). Furthermore, temporal asynchrony in sowing and harvesting schedules generates a recovery window for subordinate species, particularly shade-tolerant, slower-establishing crops such as soybean, enabling them to offset early competitive losses and even achieve yield gains (Li et al., 2001). The selection effect enhances the yield advantage of intercropping systems by increasing the yield of dominant crops (i.e., competitively superior), while the complementarity effect elevates the overall system advantage through interspecific complementarity (e.g., niche differentiation in resource use) and yield improvement of subordinate crops. The dynamic balance between interspecific complementarity and competition underpins the high yield and long-term stability of intercropping systems.

Among various intercropping patterns, cereal–legume intercropping system is one of the most widely adopted and intensively investigated classic combinations globally. In this system, cereal crops (e.g., maize) ensure staple food supply, while legume crops (e.g., soybean) improve soil fertility through rhizobial nitrogen fixation and provide high-quality protein for human and animal nutritional needs. This complementarity enables simultaneous increases in grain and protein yields while substantially reducing synthetic fertilizer inputs, resulting in significantly superior economic and ecological benefits compared with other intercropping configurations (Salinas-Roco et al., 2024). Nevertheless, previous research has disproportionately focused on the “tall-dominant-crop selection effect”, largely attributing system yield advantage to competitive dominance by the taller component alone and widely regarding it as the core underlying mechanism (Ahmed et al., 2000). The grain maize/soybean intercropping system (GM/S) is a typical example. In this system, grain maize dominates due to vigorous growth, tall stature, and superior light capture, contributing the majority of system yield, while soybean, constrained by its short stature and lower competitive ability for aboveground resources, suffers severe yield loss (Prasad and Brook, 2005; Li et al., 2021c). This research paradigm, centered on dominant crops, gives limited consideration to the compensatory growth potential of subordinate soybean. The fresh maize/soybean intercropping system (FM/S) provides an ideal platform to address this research gap. Unlike grain maize harvested at physiological maturity in late September, fresh maize is commonly harvested at the milk-to-dough stage in mid-to-late August, while soybean is harvested in early-to-mid September. This produces a natural phenological asynchrony of approximately 20-30 days, with greatly alleviated interspecific competition from fresh maize. During this period, soybean experiences greatly relieved competitive constraints on light, water, and nutrients. This condition may facilitate improved photoassimilate partitioning toward seeds, enhanced nitrogen uptake and remobilization, higher 100-seed weight and a lower shriveled grain rate. Consequently, soybean achieves recovery and compensatory growth. Thus, conventional cereal-legume intercropping theories mainly focus on spatial complementarity, including canopy stratification and root niche partitioning during crop co-growth stages, which is regarded as the core source of intercropping advantages. However, existing theories largely ignore the independent and synergistic value of temporal niche differentiation. Specifically, how post-dominant-crop harvesting temporal compensation regulates subordinate crop recovery and further couples with spatial complementarity to reshape system productivity balance remains unclear and lacks systematic theoretical elucidation. Therefore, the FM/S intercropping system, which integrates both spatial niche partitioning and late-stage temporal compensation conditions, provides a novel and feasible model to explore the coupled “spatial complementarity + temporal compensation” mechanism. Clarifying this ecological process is the core scientific objective of the present study.

Based on the current research status, we formulate the following hypotheses: (1) Under identical row ratio and planting density configuration, the FM/S system may trigger obvious soybean recovery and strengthen the system complementarity effect by providing soybean with a post-harvest sole growth period exceeding 20 days, we expect that soybean partial land equivalent ratio (PLER), overall system LER and net effect will be higher in FM/S relative to the GM/S system. (2) The temporal niche differentiation arising from the early harvest of fresh maize will enable both the selection effect and complementarity effect in the FM/S system to remain positive and proportionally balanced. In contrast, due to the absence of a late compensatory growth window for soybean in the GM/S system, the complementarity effect may become weak or negative, such that the yield advantage of this system relies heavily on the selection effect. This study conducted a three-year field experiment from 2023 to 2025 with the following objectives: (1) compare the differences in yield, LER, net effect (NE), and interspecific competitiveness between the FM/S and GM/S systems under varying row ratio and planting density configurations, and identify their respective optimal configurations; (2) quantify both the absolute magnitudes and relative contributions (i.e., proportional shares) of selection effect and complementarity effect in each system; (3) clarify whether the “spatial complementarity + temporal compensation” dual driven paradigm shows advantages over conventional intercropping models that rely merely on the selection effect.

## 2 Materials and methods

### 2.1 Experimental site

Field experiments were conducted from 2023 to 2025 at the Experimental Station of Shanxi Agricultural University (112°52´ E, 38°02´ N, altitude 1270 m) in Taiyuan City, Shanxi Province, China. The site is located in the north temperate zone and experiences a temperate continental monsoon climate, with a mean annual temperature of 7.1 °C, mean annual precipitation of 450 mm, a frost-free period of 120 days, and an effective accumulated temperature ≥10 °C of 2600 °C.d The climate is cool with a large diurnal temperature variation. The surface soil layer (0–20 cm) of the experimental site was sandy loam, contained 16.50 g kg^−1^ organic matter, 1.09 g kg^−1^ total nitrogen, 55.12 mg kg^−1^ available nitrogen, 14.63 mg kg^−1^ available phosphorus, 161.09 mg kg^−1^ available potassium, and had a pH of 8.02. The experiment was conducted under rainfed conditions (i.e., no supplemental irrigation). The initial preceding crop in the experimental field was maize. To ensure consistent preceding crop conditions across experimental plots each year, the field was divided into two equal sections that were rotated annually: one section was used for the current experiment, and the other was planted with maize. Annual precipitation during the three-year field trial and the regional 20-year mean annual precipitation are presented in **Figure 1**.

**Figure 1:**
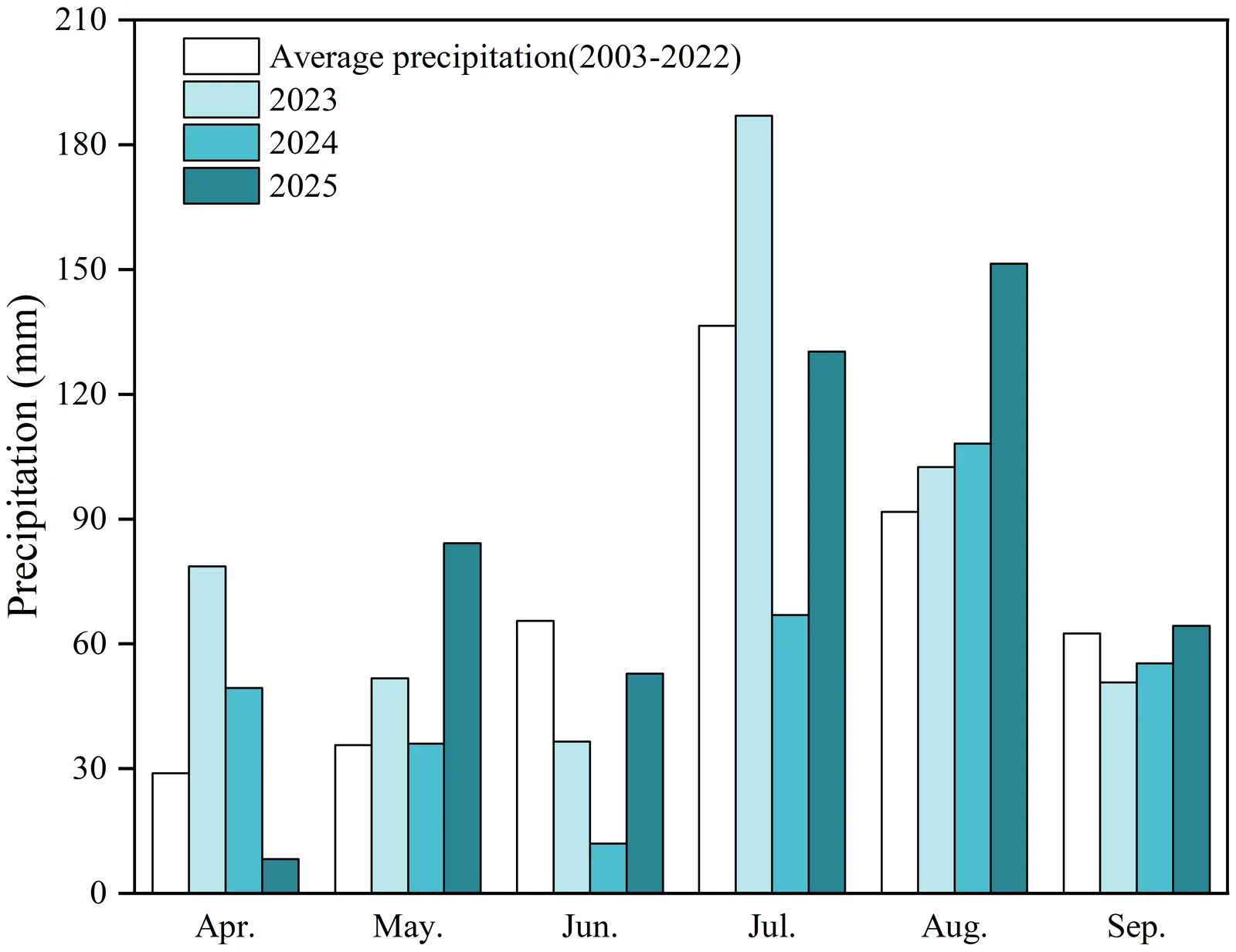
Long-term average (20-year) and experimental period (2023–2025) precipitation at the experimental site.

### 2.2 Experimental design

The experiment employed a three-factor randomized complete block design. Factor 1 was the intercropping system: fresh maize/soybean (FM/S) and grain maize/soybean (GM/S). Factor 2 was the two row configurations: 4:2 (i.e., four rows of fresh maize or grain maize alternating with two rows of soybean, denoted as 4FM2S or 4GM2S) and 4:4 (i.e., four rows of fresh maize or grain maize alternating with four rows of soybean, denoted as 4FM4S or 4GM4S). Factor 3 was the planting density of intercropped fresh maize or grain maize, with three levels based on the respective sole cropping densities: low density (LD; −20% of sole cropping density: 42,000 plants ha^−1^ for fresh maize, 48,000 plants ha^−1^ for grain maize), medium density (MD; −10%: 47,250 plants ha^−1^ for fresh maize, 54,000 plants ha^−1^ for grain maize), and high density (HD; 0%: 52,500 plants ha^−1^ for fresh maize, 60,000 plants ha^−1^ for grain maize). Within the 4FM2S and 4GM2S intercropping treatments, soybean density was fixed at 56,300 plants ha^−1^; within the 4FM4S and 4GM4S treatments, it was 93,300 plants ha^−1^.

Sole-crop controls included fresh maize (SFM, 52,500 plants ha^−1^), grain maize (SGM, 60,000 plants ha^−1^), and soybean (SS, 154,000 plants ha^−1^). A total of 15 treatments were established in this experiment, each with three replicates, resulting in 45 experimental plots. Plot area was standardized according to planting configuration: 40.5 m^2^ (8.1 m × 5 m) for 4:2 intercropping, and 49.5 m^2^ (9.9 m × 5 m) for 4:4 intercropping. Sole-crop plots were area-matched to their respective intercropping counterparts (i.e., 40.5 m^2^ for those assigned to the 4:2 system and 49.5 m^2^ for those in the 4:4 system)—to ensure comparability of land area and resource availability across cropping systems. The tested cultivars were fresh maize ‘Wannuo 2000’, grain maize ‘Chengxin 16’, and soybean ‘Jindou No. 1’. Detailed field layouts for sole cropping and intercropping treatments are presented in **Table 1**, **Figure 2**.

**Table 1:** Row and plant spacing configurations of grain maize, fresh maize, and soybean under sole and intercropping systems during 2023–2025.

Grain maize (fresh maize)/soybean row ratio	Grain maize (fresh maize) density	Grain maize		Fresh maize		Soybean	
		Plant spacing cm	Row spacing cm	Plant spacing cm	Row spacing cm	Plant spacing cm	Row spacing cm
4:2	LD	31	40	35	40	13	30
	MD	28	40	31	40	13	30
	HD	25	40	28	40	13	30
4:4	LD	25	40	29	40	13	30
	MD	22	40	26	40	13	30
	HD	20	40	23	40	13	30
Sole cropping		28	60	32	60	13	50

4:2 and 4:4 denote four rows of grain maize (or fresh maize) alternated with two rows of soybean, and four rows of grain maize (or fresh maize) alternated with four rows of soybean, respectively; LD, MD, and HD represent low, medium, and high planting densities of grain maize (or fresh maize), corresponding to 20% below, 10% below, and equal to their respective sole cropping densities.

**Figure 2:**
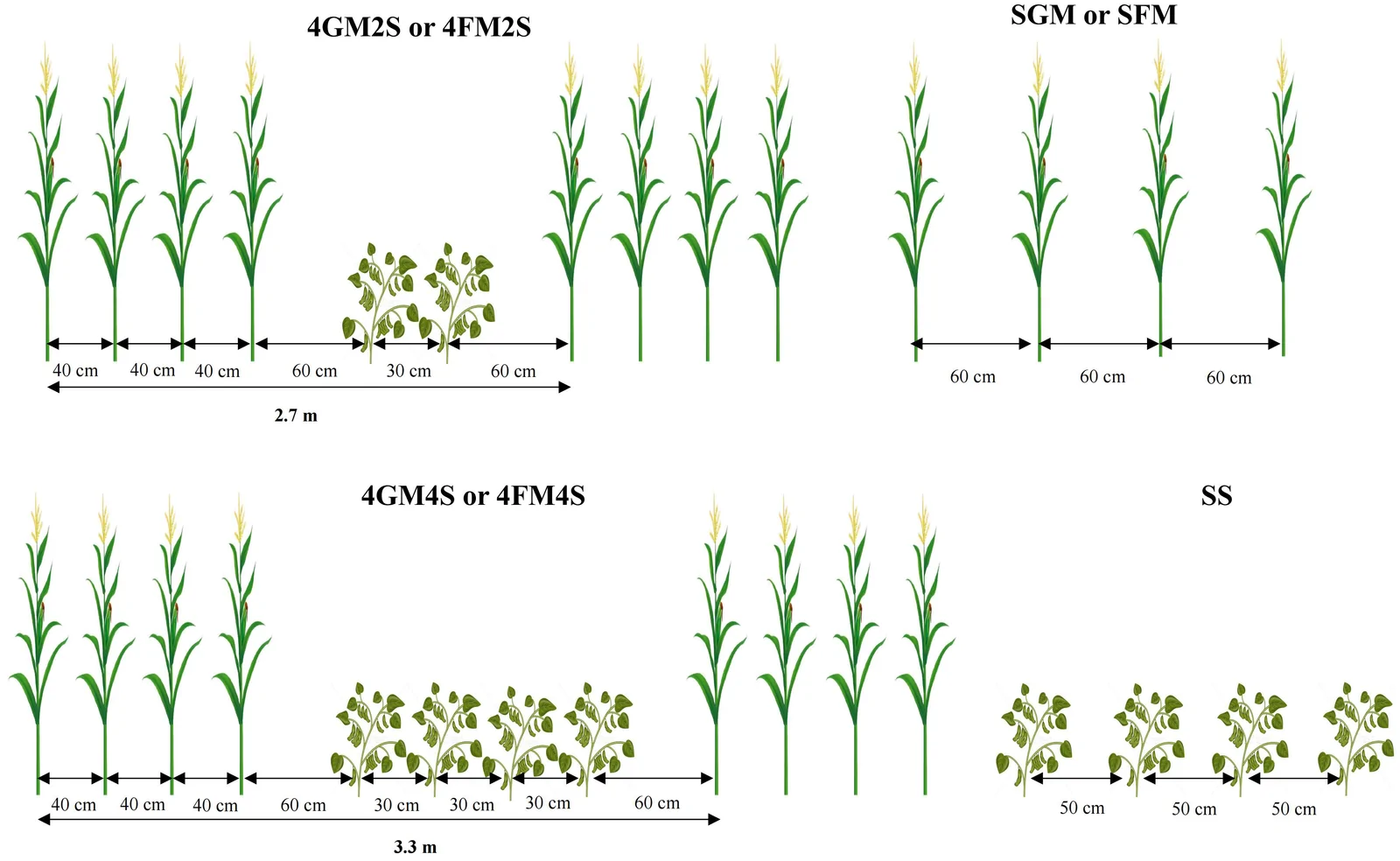
Field layout of intercropping and sole cropping patterns for grain maize, fresh maize, and soybean (2023–2025). 4GM2S or 4FM2S, four rows of grain maize (or fresh maize) intercropped with two rows of soybean; 4GM4S or 4FM4S, four rows of grain maize (or fresh maize) intercropped with four rows of soybean; SGM or SFM, sole cropping of grain maize or fresh maize; SS, sole cropping of soybean.

Basal fertilizer was uniformly applied to all experimental plots before sowing at rates of 80 kg N ha^−1^, 52 kg P_2_O_5_ ha^−1^, and 80 kg K_2_O ha^−1^. These rates were supplied by urea (46% N), single superphosphate (16% P_2_O_5_) and potassium sulfate (50% K_2_O), respectively. On this basis, both grain maize and fresh maize received an additional topdressing of 120 kg N ha^−1^ in both sole and intercropping systems. Topdressing was manually applied in furrows between rows at the V11–V13 growth stage for grain maize and at the V8–V10 stage for fresh maize, aligning with their respective peak nitrogen demand periods. Consequently, the total nitrogen application rate for grain maize and fresh maize during the entire growing period was 200 kg N ha^−1^. In 2023, fresh maize, soybean, and grain maize were sown simultaneously on May 12 and harvested on August 29, September 19, and October 13, respectively. in 2024, the same cultivars were sown simultaneously on May 2 and harvested on August 22, September 18, and October 10, respectively; in 2025, sowing occurred on April 25, with harvests on August 22, September 10, and September 30, respectively. After fresh maize harvesting, all maize straw was completely removed from the field to eliminate shading and ensure full light interception of soybean at its late growth stage. Crucially, both the spatial planting configuration and the complete fertilization protocol, including basal application rates and top-dressing timing and rates, were strictly maintained across all three years to ensure experimental continuity and temporal comparability.

### 2.3 Sample collection and methods

Yield sampling was conducted at the maturity stage of grain maize, fresh maize, and soybean. Each intercropping plot contained three planting strips; the central strip was selected for harvest, and all plants within this strip were collected from a uniform row length of 4 m (the total designed row length was 5 m per plot). In sole cropping plots, four rows in the central area of each plot were selected, with a continuous 4 m length harvested from each row (i.e., the inner 4 m of the 5 m row length). Grain yields were adjusted to 14% moisture content, fresh maize yields were calculated as marketable fresh ears, and soybean yields were adjusted to 13% moisture content.

### 2.4 Calculations

#### 2.4.1 Land equivalent ratio

The land equivalent ratio (LER) is the most fundamental metric for evaluating resource use efficiency in intercropping systems. It quantifies the yield advantage of intercropping relative to sole cropping (Equation 1) (Vandermeer, 1989).

(1) $$\text{LER}={\text{PLER}}_{a}+{\text{PLER}}_{b}=\frac{Y_{ia}}{Y_{sa}}+\frac{Y_{ib}}{Y_{sb}}$$

Where PLER_a_ and PLER_b_ represent partial land equivalent ratios of crop a and b, respectively; *Y*_sa_ and *Y*_sb_ are the yields of crop a and crop b under sole cropping, respectively; *Y*_ia_ and *Y*_ib_ are the yields of crop a and crop b under intercropping, respectively. If LER > 1, the intercropping system exhibits a yield advantage, reflecting more efficient land use and stronger system-level competitiveness; If LER< 1, the intercropping system suffers a yield penalty, indicating strong interspecific competition that reduces resource-use efficiency and results in weaker overall competitiveness relative to sole cropping.

#### 2.4.2 Competitive ability

Competitive Ratio (CR) and Aggressivity (A) were used to assess the competitive relationships in the intercropping system (Equations 2, 3). CR quantifies the relative competitive ability of two crops, reflecting the competitive capacity of one crop relative to the other (Ma et al., 2024).

(2) $${\text{CR}}_{a-b}=\frac{{\text{PLER}}_{a}}{{\text{PLER}}_{b}}\times \frac{P_{b}}{P_{a}}$$

Where CR_a−b_ is the competitive ratio of crop a relative to crop b; PLER_a_ and PLER_b_ are defined in Equation (1); *P*_a_ and *P*_b_ represent the proportional planting areas of crop a and crop b in the intercropping system, respectively. In the 4:2 planting pattern, the area proportions are 0.667 for grain maize (or fresh maize) and 0.333 for intercropped soybean; in the 4:4 pattern, they are 0.545 and 0.455, respectively. If CR_a−b_ > 1, crop a is more competitive than crop b, reflecting its dominance in light interception, water uptake, and nutrient acquisition.

A is a key indicator for quantifying the absolute competitive advantage of one crop over another in intercropping systems. By calculating the difference in relative yield per unit resource between the two crops, it directly reflects the net yield gain (or loss) attributable to the competitive dominance (or suppression) of crop a relative to crop b under shared resource conditions (Gao et al., 2024).

(3) $$A_{a-b}=\frac{Y_{ia}}{Y_{sa}\times P_{a}}-\frac{Y_{ib}}{Y_{sb}\times P_{b}}$$

Where A_a−b_ represents the absolute competitiveness of crop a relative to crop b; *Y*_sa_, *Y*_sb_, *Y*_ia_, *Y*_ib_, *P*_a_ and *P*b are as defined in Equations (1) and (2). When A_a−b_ > 0, crop a has an absolute competitive advantage over crop b, quantifying the absolute yield gain attributable to its superior resource acquisition under interspecific competition. The magnitude reflects the intensity of this dominance: larger values signify stronger competitive suppression of crop b. A negative A_a−b_ indicates that crop a is competitively suppressed by crop b, with increasingly negative values corresponding to greater yield loss and reduced functional capacity in resource acquisition.

As dimensionless indices, Competitive ratio (CR) and aggressivity (A) were derived using internally consistent yield data within each system: fresh-ear yield for FM/S and grain yield for GM/S. Cross-system comparisons of CR and A reflect relative interspecific competitive patterns under respective economic-yield benchmarks.

#### 2.4.3 Intercropping effect

The intercropping effect was quantified using the complementarity effect (CE), selection effect (SE), and net effect (NE) (Equations 4–6) (Loreau and Hector, 2001). NE is a quantitative indicator of the overall performance of intercropping systems, defined as the difference between the actual yield of intercropped crops and their expected yield under sole cropping at the same planting proportion. Conceptually, NE integrates the net outcome of interspecific facilitation and competition.

(4) $$\text{NE}=\left(Y_{ia}+Y_{ib}\right)-\left(Y_{sa}\times P_{a}+Y_{sb}\times P_{b}\right)$$

A positive NE (NE > 0) signifies a net yield advantage of intercropping over sole cropping, reflecting that interspecific facilitation outweighs competition. Moreover, the magnitude of NE is directly indicative of the strength of this advantage, the larger the NE value, the stronger the interspecific facilitation and the greater system-level efficiency. Conversely, a negative NE (NE< 0) indicates a yield penalty, where competitive interference exceeds any beneficial interactions, resulting in a negative effect.

CE directly reflects the net outcome of interspecific facilitation—i.e., the degree to which positive interactions mitigate the negative impacts of interspecific competition.

(5) $$\text{CE}=\left[\left(\frac{Y_{ia}}{Y_{sa}}-P_{a}\right)+\left(\frac{Y_{ib}}{Y_{sb}}-P_{b}\right)\right]\times \left(\frac{Y_{sa}+Y_{sb}}{2}\right)$$

A positive CE (CE > 0) indicates significant interspecific facilitation, enabling the subordinate crop to compensate for competitive yield losses and even achieve a yield gain. A negative CE (CE< 0) suggests insufficient interspecific facilitation, meaning subordinate crops cannot compensate for competition-induced yield losses, resulting in intensified yield reduction.

SE is an indicator quantifying the yield advantage gained by the dominant crop in intercropping as a result of interspecific competition.

(6) SE=NE−CE

When SE > 0, the high-yielding dominant crop benefits in the intercropping system; the larger the value, the greater the yield benefit of the dominant crop. When SE< 0, the dominant crop fails to realize its inherent yield potential under intercropping. Here, *Y*_sa_, *Y*_sb_, *Y*_ia_, *Y*_ib_, *P*_a_, and *P*_b_ are as defined in Equations (1) and (2).

Fresh-ear yield was adopted for the FM/S system, and grain yield for the GM/S system. These indices were interpreted only within each intercropping system, and cross-system comparisons of their absolute values were not conducted.

#### 2.4.4 The contribution ratios of CE and SE

The contribution ratios of CE and SE were calculated using (Equations 7, 8).

(7) $${\text{CE}}_{ratio}=\frac{\text{CE}}{\text{NE}}\times 100\%$$

(8) $${\text{SE}}_{ratio}=\frac{\text{SE}}{\text{NE}}\times 100\%$$

Where CE_ratio_ and SE_ratio_ represent the contributions of CE and SE to NE, respectively.

#### 2.4.5 Theoretical sole cropping yield

The theoretical sole cropping yield is a virtual weighted yield rather than a field-measured yield. It is calculated from the actual sole-crop yield of each component crop and their corresponding planting area proportions in the intercropping system. This indicator enables comparisons between intercropping and sole-cropping under an identical total land area, we can evaluate the yield advantages or disadvantages of the intercropping system (Equation 9).

(9) $$Y_{\text{theoretical}}=Y_{\text{sa}}\times P_{\text{a}}+Y_{\text{sb}}\times P_{\text{b}}$$

Where *Y*_theoretica_l refers to the theoretical sole-cropping yield; *Y*_sa_, *Y*_sb_, *P*_a,_ and *P*_b_ are as defined in Equations 1, 2.

#### 2.4.6 Economic benefit calculation

The economic benefit was adopted to evaluate the economic performance of different cropping patterns in this study. It was calculated from the actual yield of each crop and the local in-season purchasing price in the corresponding experimental year. It should be noted that the economic benefit in the present study only represents the total crop output revenue, and agricultural production input costs were not deducted.

### 2.5 Statistical analysis

Experimental data were organized using Microsoft Excel 2021 and statistically analyzed with SPSS 22.0 (IBM Corp.). A hierarchical statistical approach was adopted in this study. Row ratio and planting density were treated as fixed effects, whereas experimental year and block were assigned as random effects. Two-way analysis of variance (ANOVA) was conducted to assess the main and interactive effects of row ratio and planting density on crop yield, LER, CR, A, CE, SE, and NE within each intercropping system. Following this, the optimal planting configuration screened from each system was compared hierarchically to examine inter-system differences. Tukey’s HSD test was used for multiple comparisons among different treatments, with a significance level set at p< 0.05. All results are expressed as mean ± standard error. All experimental figures were plotted using Origin 2021 (OriginLab).

## 3 Results

### 3.1 Crop yield

The different row ratio and planting density configurations significantly influenced the yields of grain maize and fresh maize (including border and inner rows) as well as the yields of intercropped soybean (*P* < 0.05, **Figure 3**). Relative to theoretical sole cropping yield, three-year mean yields of intercropped grain maize and fresh maize increased by 25.97% (range: 15.74–38.14%) and 17.29% (range: 12.45–26.34%), respectively. In contrast, intercropped soybean yield declined significantly by 37.32% (range: 25.36–50.45%) in the GM/S system and by 16.07% (range: 7.50–23.39%) in the FM/S, compared with their respective theoretical soybean sole cropping yield (*P* < 0.05). At the same row ratio, grain maize yield increased with increasing planting density. Fresh maize yield under the 4:2 row ratio followed a similar positive response to density, whereas under the 4:4 row ratio, it exhibited a unimodal response—peaking at medium densities before declining at high densities. Intercropped soybean yield decreased significantly with increasing planting density of either grain maize or fresh maize (*P* < 0.05). At the same planting density, both grain maize and fresh maize yields were significantly higher under the 4:2 row ratio than under the 4:4 row ratio, while soybean yield exhibited the opposite trend (P<0.05). Yields of grain maize, fresh maize and soybean exhibited obvious year-to-year fluctuations across the two intercropping systems, with the highest values observed in 2023, followed by 2025, and the lowest in 2024. The yields were closely related to interannual precipitation. Specifically, cumulative precipitation during the growing season was significantly lower in 2024 than in 2023 and 2025 (**Figure 1**). Fresh maize yield decreased more sharply than grain maize yield from 2023 to 2024, with average reductions of 32.26% and 17.22%, respectively. In contrast, fresh maize yield rebounded more strongly than grain maize yield from 2024 to 2025, increasing by 33.57% versus only 3.40%.

**Figure 3:**
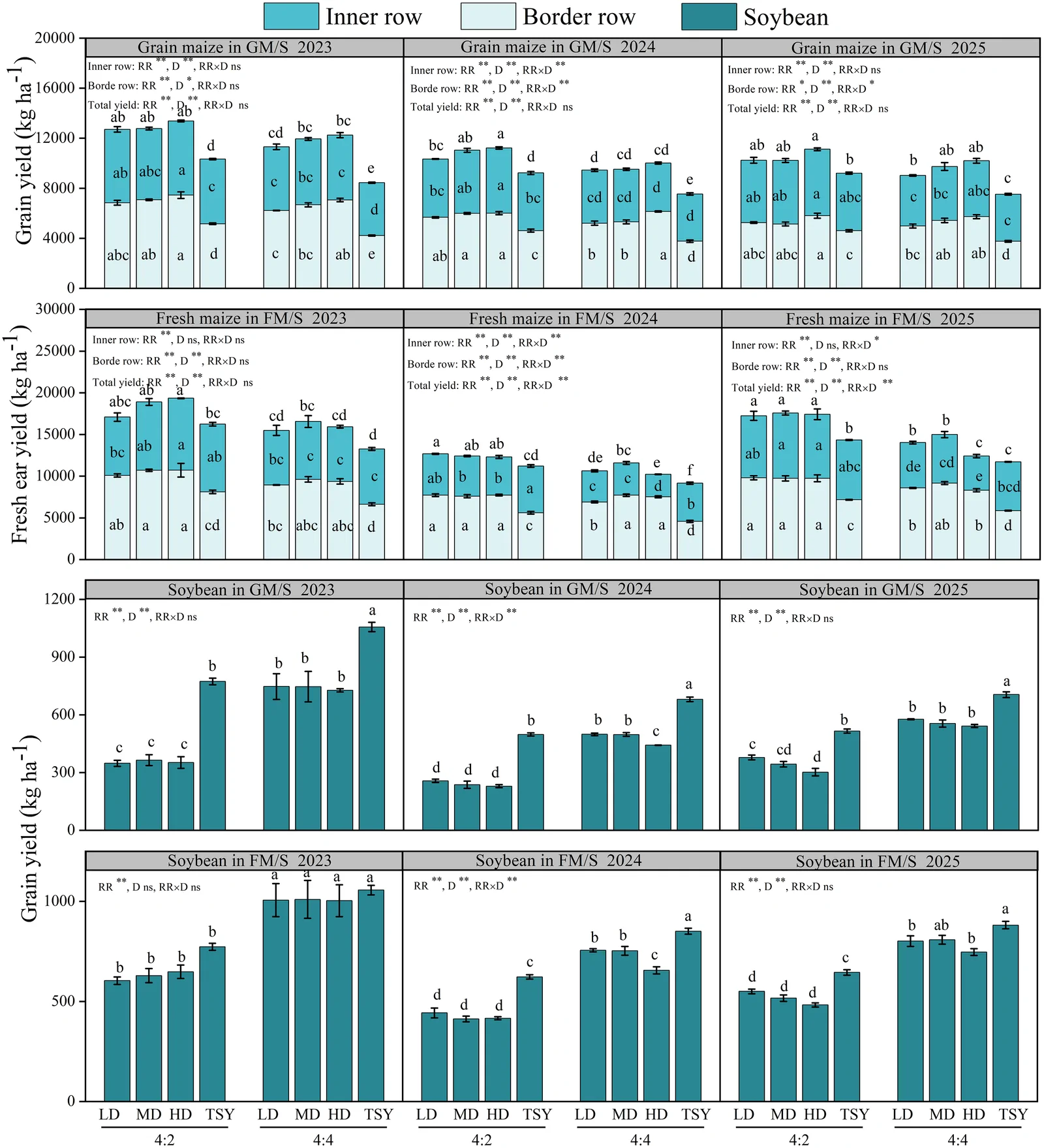
Response of border and inner row yields of grain maize and fresh maize, and soybean yield, to row ratio and planting density of grain maize (or fresh maize) with soybean (2023–2025). Theoretical sole cropping yield (TSY) refers to the weighted yield of grain maize (or fresh maize) and soybean under sole cropping, calculated according to their respective planting proportions in the intercropping system. RR, grain maize (or fresh maize)/soybean row ratio; D, grain maize (or fresh maize) planting density; other abbreviations are the same as those in **Table 1**. Values are mean ± SE (n=3). * and ** indicate P<0.05 and P<0.01, respectively; ns, no significant difference. Different lowercase letters indicate significant differences at p<0.05. Values are mean ± SE (n=3). * and ** indicate P < 0.05 and P < 0.01, respectively; ns, no significant difference. Different lowercase letters indicate significant differences at P < 0.05.

Border rows played a critical role in yield enhancement. Over the three-year average (**Figure 3**), border rows of grain maize contributed 74.44% of the total yield increase (range: 70.33–80.35%), while in fresh maize, their contribution exceeded the overall yield gain—reaching 123.56% (range: 100.25–183.87%). A contribution value above 100% indicates that border-row gain offsets yield losses from inner rows within the same plot. This implies that, in fresh maize, the yield gain attributable to border rows alone surpassed the net yield increase observed across the entire plot, highlighting a disproportionately strong border effect. In both GM/S and FM/S systems, inner-row yields were consistently lower than border-row yields (*P* < 0.05). However, their performance relative to the theoretical sole cropping yield differed markedly: inner-row grain maize yields exceeded the theoretical sole cropping yield, whereas inner-row fresh maize yields fell below it across all observed conditions.

### 3.2 The LER based on yield

The two intercropping systems (GM/S and FM/S) differed in land equivalent ratio (LER) (**Figure 4**). Over the three-year average, FM/S consistently achieved higher LER values than GM/S except under the 4:4 high-density configuration. For GM/S, LER was consistently greater than 1 under the 4:4 row ratio but below 1 under the 4:2 row ratio, peaking at 1.071 under the 4:4 high-density configuration. In contrast, the mean LER of FM/S exceeded 1 across all configurations, with its highest value (1.107) observed under the 4:4 medium-density treatment. At the same row ratio, LER increased with planting density in GM/S, whereas in FM/S it followed a unimodal response—rising to a peak before declining with further density increases. Under the same planting density, the LER of the 4:4 row ratio was higher than the 4:2 row ratio across all treatments, except under high-density conditions in FM/S, where the 4:2 configuration outperformed the 4:4 configuration. Compared with 2024, the LER of GM/S and FM/S increased by 5.35% (range: 2.63%–8.19%) and 6.23% (range: –2.91%–13.27%) in 2023, respectively, and by 3.87% (range: 0.62%–7.24%) and 6.46% (range: 0.33%–13.02%) in 2025. This indicates that the seasonal drought in 2024 visibly reduced land use efficiency in both intercropping systems, whereas the more favorable rainfall conditions in 2023 and 2025 (characterized by higher total precipitation and improved temporal distribution, **Figure 1**) obviously improved resource-use efficiency in GM/S and FM/S.

**Figure 4:**
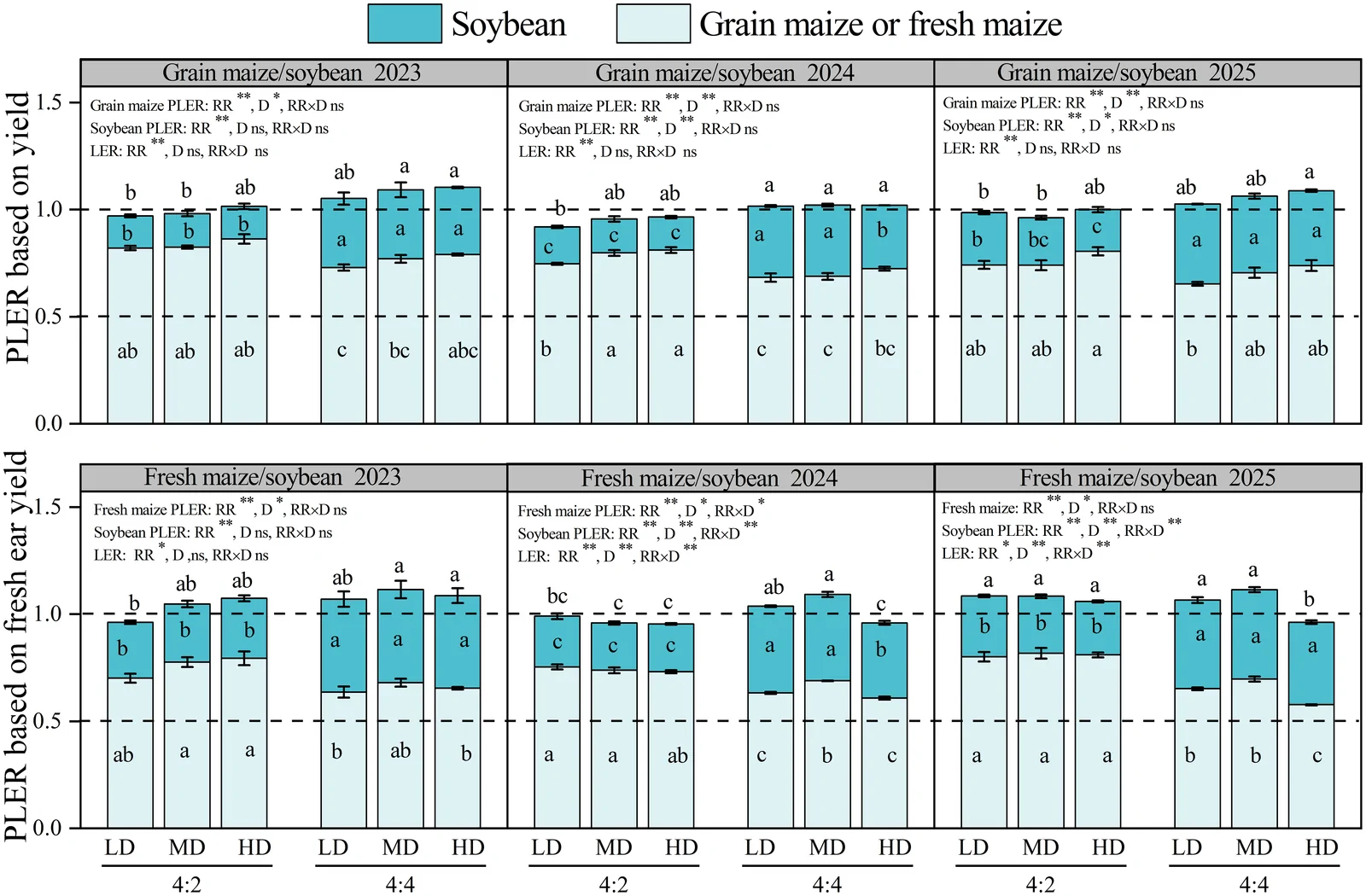
Partial land equivalent ratios (PLER) of grain maize, fresh maize, and soybean, and land equivalent ratio (LER) of the intercropping systems (GM/S and FM/S) (2023–2025). LER is the sum of the two corresponding PLER values for each system (grain maize + soybean for GM/S; fresh maize + soybean for FM/S). The dashed line indicates LER = 0.5 and LER = 1. Other abbreviations are the same as those in **Table 1**, **Figure 3**. Values are mean ± SE (n=3). * and ** indicate P < 0.05 and P < 0.01, respectively; ns, no significant difference. Different lowercase letters indicate significant differences at P < 0.05.

The partial land equivalent ratios (PLER) of both grain maize and fresh maize were greater than 0.5 (*P* < 0.05, **Figure 4**), indicating their competitive dominance within their respective intercropping systems; In contrast, the PLER of soybean was below 0.5 in both systems, reflecting its subordinate role under interspecific competition. In GM/S and FM/S, the PLER of the main crops (grain maize and fresh maize) showed no significant difference between systems. In contrast, the PLER of soybean differed markedly between the two systems (*P* < 0.05): intercropped soybean exhibited a significantly higher PLER in FM/S than in GM/S, with an average increase of 32.80% (range: 21.79–50.10%).

### 3.3 Crop competitiveness

The competitive ratio (CR) and aggressivity (A) of grain maize relative to soybean were significantly higher than those of fresh maize relative to soybean across all three years (**Figure 5**). These dimensionless indices reflect relative interspecific competitive patterns under their respective economic-yield benchmarks. Across the three-year period, the CR of grain maize relative to soybean averaged 2.07, while that of fresh maize relative to soybean averaged 1.43—both values exceeding the threshold of 1. Similarly, the A of grain maize and fresh maize relative to soybean averaged 0.62 and 0.34, respectively—both distinctly positive. These results indicate that both maize types competitively dominate soybean in their respective intercropping systems. Under the same row ratio, both the CR and A of grain maize relative to soybean increased monotonically with increasing plant density. In contrast, fresh maize exhibited divergent density-dependent responses depending on the row configuration: under the 4:2 row ratio, its CR and A relative to soybean increased consistently with density, mirroring the pattern observed for grain maize; however, under the 4:4 row ratio, both metrics peaked at an intermediate density and subsequently declined. Furthermore, at the same density level, both CR and A were higher under the 4:2 row ratio than under the 4:4 row ratio. Compared with 2023, the average CR and A of grain maize relative to soybean decreased by 10.54% and 14.40%, respectively, in 2024, and declined by 28.15% and 34.30% in 2025. In contrast, the CR and A of fresh maize relative to soybean increased by 12.62% and 32.88% in 2024, and by 5.59% and 20.66% in 2025.

**Figure 5:**
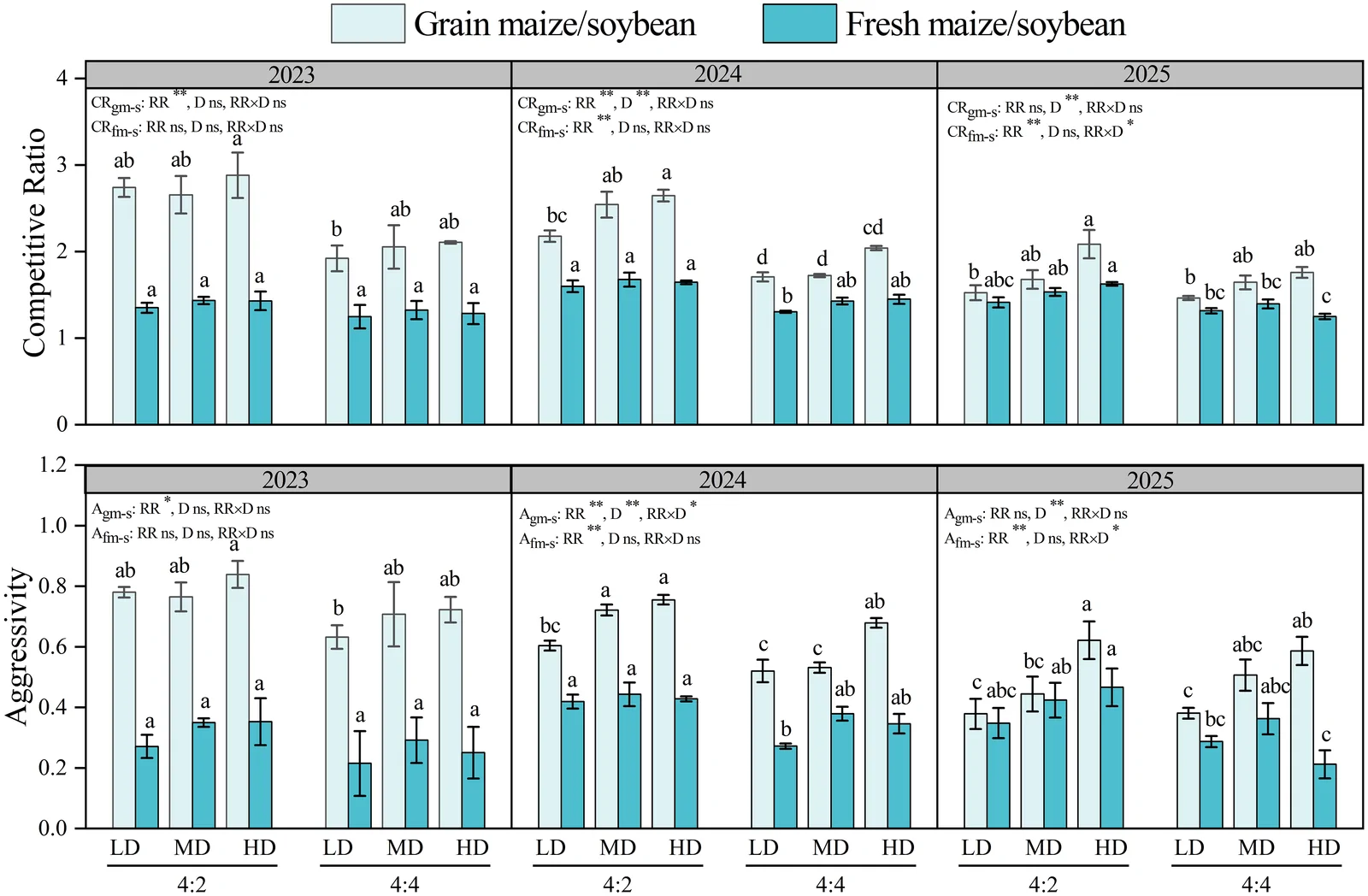
Competitive ratio and aggressivity of grain maize (or fresh maize) relative to soybean (2023–2025). CRgm-s, CRfm-s, Agm-s, and Afm-s represent the competitive ratio of grain maize to soybean, the competitive ratio of fresh maize to soybean, the aggressivity of grain maize to soybean, and the aggressivity of fresh maize to soybean, respectively. Other abbreviations are the same as those in **Table 1**, **Figure 3**. Values are mean ± SE (n=3). * and ** indicate P < 0.05 and P < 0.01, respectively; ns, no significant difference. Different lowercase letters indicate significant differences at P < 0.05.

### 3.4 Intercropping effect based on yield

Averaged across three years, the net effect (NE) and selection effect (SE) were consistently positive in both GM/S and FM/S intercropping systems (**Figure 6**). In contrast, the complementarity effect (CE) showed system- and configuration-dependent patterns: CE remained positive across all row ratios in FM/S, whereas in GM/S it was negative under the 4:2 row ratio but positive under the 4:4 row ratio (*P* < 0.05).

**Figure 6:**
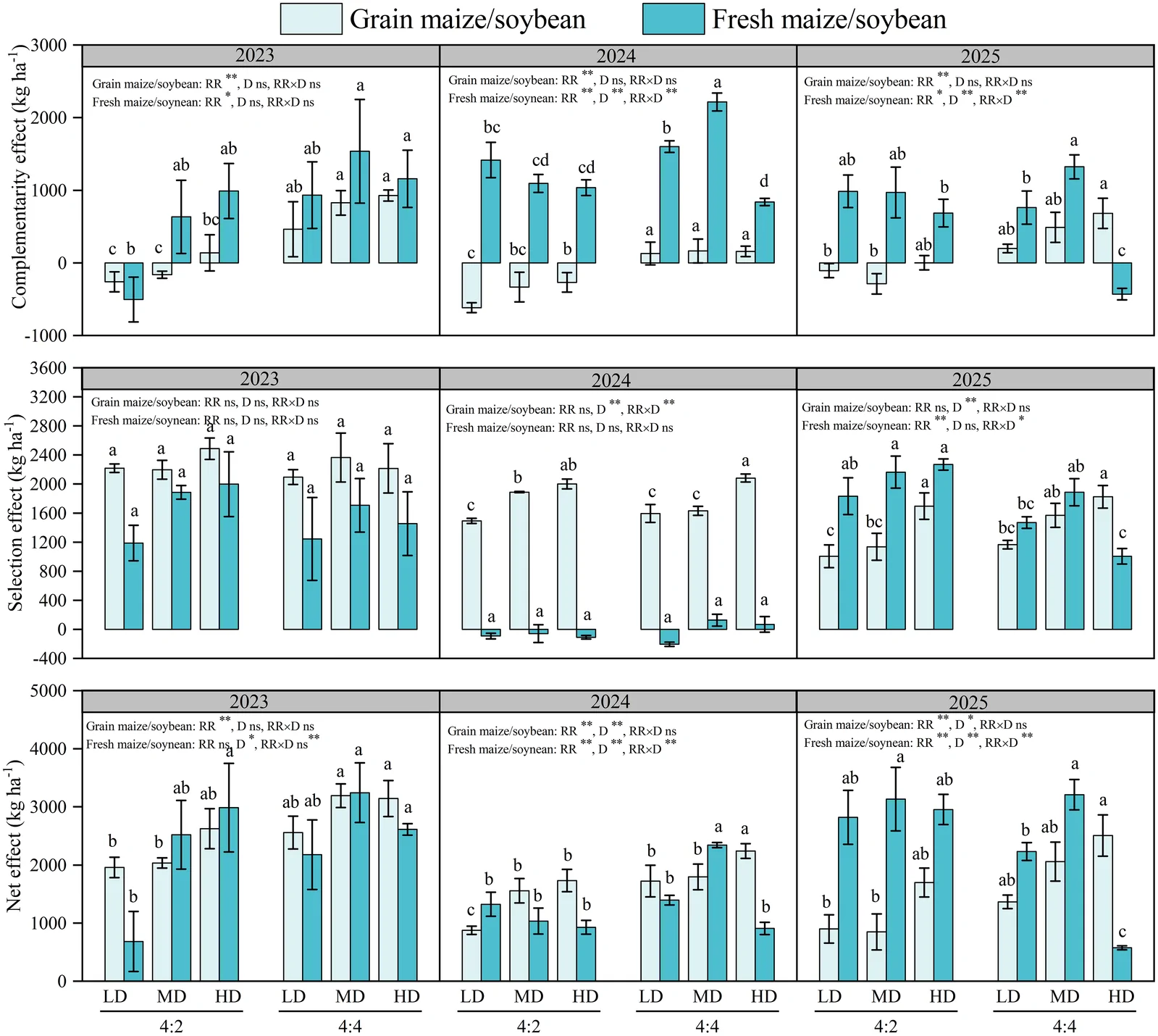
Net effect, selection effect, and complementarity effect of grain maize (or fresh maize)/soybean intercropping systems (2023–2025). Values are mean ± SE (n=3). * and ** indicate P < 0.05 and P < 0.01, respectively; ns, no significant difference. Different lowercase letters indicate significant differences at P < 0.05.

In terms of interannual variability, SE displayed contrasting temporal dynamics between the two systems: from 2023 to 2025, SE in GM/S declined steadily, whereas SE in FM/S first decreased and then increased, with substantially greater interannual amplitude than in GM/S. In contrast, CE showed opposing responses: relative to 2024, CE in FM/S decreased by 36.11% in 2023 and by 53.28% in 2025, whereas CE in GM/S increased markedly—by 148.51% in 2023 and by 64.79% in 2025. For NE, compared with 2024, GM/S exhibited an average increase of 62.05% in 2023 and no significant change in 2025; FM/S reached its lowest NE value in 2024, with no significant difference between 2023 and 2025, representing increases of 99.93% and 99.09% relative to 2024, respectively.

### 3.5 Proportions of complementarity and selection effects in intercropping systems

GM/S and FM/S exhibited fundamentally distinct dominance patterns between the CE and SE (*P* < 0.05, **Figure 7**). A three-year mean analysis revealed that GM/S was consistently dominated by the SE, which accounted for 98.12% of the NE on average (range: 77.60%–126.36%). A positive CE occurred only under the 4:4 row ratio configuration, reaching a maximum contribution of 22.40%—indicating that the yield advantage of GM/S was overly dependent on SE. FM/S exhibited a balanced contribution of CE and SE to the net effect, with three-year mean contributions of 45.32% (range: 38.24%–57.70%) and 54.68% (range: 42.30%–61.73%), respectively. Notably, the maximum proportion of CE in FM/S (57.70%) was substantially higher than that in GM/S (22.40%), indicating that FM/S is more capable of achieving efficient resource use through a positive CE.

From an interannual perspective, the effect ratios of both GM/S and FM/S intercropping systems differed markedly with annual water conditions (**Figures 1**, **7**). In 2023, under adequate water availability, the complementarity effect ratio (CE_ratio_) of GM/S was 8.76% (with the selection effect accounting for 91.34%), indicating a selection-effect-dominated system; for FM/S, the CE_ratio_ was 44.86% (with the selection effect accounting for 55.14%), and the two effects were relatively balanced. Under severe drought in 2024, the GM/S system not only lost its positive CE but displayed a net negative contribution (−16.15%), with the selection effect accounting for 116.15%, further strengthening the dominance of SE; conversely, the FM/S system exhibited a pronounced shift toward complementarity-driven overyielding, with CE contributing 105.99%, exceeding the total overyielding. In 2025 when water conditions recovered, the CE_ratio_ of GM/S rebounded to −4.34% (with the selection effect accounting for 104.34%) and remained SE-dominated; the FM/S system reverted to a selection-effect-dominated pattern, with CE declining sharply to 14.88%. (A negative CE contribution indicates yield loss induced by the complementarity component, while a ratio greater than 100% indicates that one component offsets the yield change caused by the other component within the overall net effect).

### 3.6 Economic benefit analysis

Significant differences in economic benefits were observed between GM/S and FM/S across 2023–2025 (**Table 2**). Over the three-year period, the mean economic benefit of FM/S configurations was 29,456.4 CNY ha^−1^ (range: 27,171.1–31,223.6 CNY ha^−1^), significantly higher than the GM/S mean of 26,320.9 CNY ha^−1^ (range: 25,404.5–27,213.5 CNY ha^−1^). This represents a relative advantage of 12.85% (range: 0.60–18.87%) for FM/S over GM/S. The average economic benefits of all configurations in GM/S and FM/S were significantly higher than that of sole soybean (13,418.5 CNY ha^−1^), and also exceeded their corresponding theoretical sole cropping levels (i.e., the weighted average economic return of sole maize and sole soybean on the same land area), by 11.81% for GM/S (range: 4.71–19.73%) and 10.84% for FM/S (range: 6.74–18.22%), respectively. These results provide robust evidence that both GM/S and FM/S intercropping systems deliver superior economic performance compared to sole cropping systems.

**Table 2:** Economic benefit analysis of grain maize, fresh maize, and soybean under sole and intercropping systems during 2023–2025.

Year	Grain maize (fresh maize)/soybean row ratio	Grain maize (fresh maize) density	Grain maize /soybean economic benefit CNY ha^-1^	Fresh maize/soybean economic benefit CNY ha^-1^
2023	4:2	LD	29312.4 ± 426.4bc	33576.0 ± 895.2bc
	4:2	MD	29561.2 ± 69.3bc	36846.5 ± 1205.0ab
	4:2	HD	30739.6 ± 792.3ab	37752.0 ± 1224.8ab
	4:2	TSC	27497.7 ± 382.0cd	33399.1 ± 621.0bc
	4:4	LD	29353.0 ± 948.1bc	33884.4 ± 989.6bc
	4:4	MD	30682.3 ± 217.7ab	35717.4 ± 1310.6b
	4:4	HD	31178.5 ± 225.9ab	34607.1 ± 415.7bc
	4:4	TSC	25653.3 ± 381.7d	30475.4 ± 453.4c
	Sole cropping grain maize (fresh maize)		32531.7 ± 389.6a	41379.5 ± 1091.8a
	Sole cropping soybean		17414.4 ± 359.9e	17414.4 ± 395.9d
2024	4:2	LD	23638.1 ± 176.8bc	24208.7 ± 329.1b
	4:2	MD	24955.9 ± 545.7bc	23580.2 ± 244.9b
	4:2	HD	25286.3 ± 434.2b	23411.1 ± 163.5b
	4:2	TSC	23108.7 ± 493.6c	22801.9 ± 594.3bc
	4:4	LD	23583.4 ± 576.7bc	22625.4 ± 117.1bc
	4:4	MD	23726.4 ± 513.5bc	24223.6 ± 122.2b
	4:4	HD	24362.3 ± 268.9bc	21331.6 ± 89.0cd
	4:4	TSC	20933.9 ± 397.2d	20683.2 ± 516.6d
	Sole cropping grain maize (fresh maize)		29044.8 ± 766.6a	28584.9 ± 571.7a
	Sole cropping soybean		11218.5± 192.5e	11218.5 ± 1236.1e
2025	4:2	LD	24347.5 ± 458.7b	32589.1 ± 765.7b
	4:2	MD	24053.0 ± 605.0b	32969.0 ± 971.2b
	4:2	HD	25614.7 ± 453.2b	32507.6 ± 483.8b
	4:2	TSC	23211.8 ± 340.5bc	28248.7 ± 119.4d
	4:4	LD	23277.1 ± 237.1bc	28644.1 ± 380.6cd
	4:4	MD	24615.3 ± 714.8b	30339.3 ± 434.5c
	4:4	HD	25489.3 ± 758.4b	25574.7 ± 43.7e
	4:4	TSC	21092.0 ± 269.2c	25207.6 ± 84.4e
	Sole cropping grain maize (fresh maize)		28997.9 ± 556.8a	36549.4 ± 262.5a
	Sole cropping soybean		11622.4 ± 244.6d	11622.4 ± 244.6f

The theoretical sole cropping (TSC) economic benefit refers to the theoretical total income calculated based on the grain maize (or fresh maize) and soybean yields under sole cropping, using the same planting proportions as in the intercropping system. Economic benefits are expressed in CNY ha^−1^, with values calculated according to local market prices in the corresponding year. Values are mean ± SE (n=3). Different lowercase letters within the same column indicate significant differences at p<0.05.

The economic benefit of GM/S increased with rising grain maize planting density, and the 4:2 row ratio yielded higher economic returns than the 4:4 row ratio at the same density. For FM/S, the economic benefit increased monotonically with density under the 4:2 row ratio, whereas under the 4:4 row ratio, it exhibited a unimodal response, peaking at medium density and declining thereafter. Notably, the maximum economic benefits for both intercropping systems were observed under the 4:2 high-density configuration (27,213.5 CNY ha^−1^ for GM/S; 31,223.6 CNY ha^−1^ for FM/S), and these economic optima differed from their corresponding LER maxima (GM/S: 4:4 high density; FM/S: 4:4 medium density). At those LER-optimal configurations, economic benefits were 27,010.0 CNY ha^−1^ and 30,093.5 CNY ha^−1^, respectively—only 0.75% and 3.76% lower than the maxima, with differences statistically non-significant (*P* > 0.05). Across experimental years, FM/S showed a 3.43% lower average economic benefit than GM/S in 2024, while FM/S exhibited higher economic benefit relative to GM/S in 2023 and 2025, with relative increases of 18.14% and 23.21%, respectively.

## 4 Discussion

### 4.1 Intercropping advantage: synergistic analysis of yield, LER, and NE

The productivity advantage of intercropping systems arises from the synergistic integration of differential yield increases, optimized resource use efficiency, and interspecific interactions (Kermah et al., 2017; Jiao et al., 2021). This study demonstrates that both GM/S and FM/S exhibit significant intercropping advantages. Specifically, intercropping consistently increased grain maize (or fresh maize) yield while decreasing soybean yield (**Figure 3**) (Li et al., 2021b; Meng et al., 2024), displaying the classic pattern of “main crop yield increase, companion crop yield decrease”, which reflected interspecific resource reallocation under shared environmental constraints. Grain maize and fresh maize, both high-efficiency C4 crops, exhibit superior light interception, spatial occupation, and nutrient-use efficiency compared with soybean. Their inherent physiological advantages, together with potential yield-promoting contributions from border-row effects (**Figure 3**), may partly contribute to yield enhancement in intercropping systems (Feng et al., 2024). Meanwhile, intercropped soybean suffers from yield reduction due to canopy shading and resource competition. However, the yield gain of main crops (grain maize and fresh maize) consistently outweighs the soybean yield loss, resulting in a net increase in overall system productivity. This pattern provides the yield basis for the observed positive intercropping advantage (Tang et al., 2024).

The land equivalent ratio (LER) further reflects the resource-use advantage of intercropping systems (Yang et al., 2024). Based on the three-year average, the LER values of both GM/S and FM/S were greater than 1 under the 4:4 row ratio configuration, demonstrating a clear land-use advantage over sole cropping under this configuration. For FM/S, the LER values across all row ratio and density configurations were greater than 1 (**Figure 4**), suggesting that FM/S can consistently achieve intercropping advantages across different population structures, which may indicate superior stability in resource partitioning and utilization efficiency compared with GM/S. The superiority of intercropping over sole cropping stems from interspecific niche complementarity, particularly in the spatiotemporal partitioning of light, heat, water, and nutrients, which enables enhanced resource capture and utilization efficiency through optimized row spacing and planting density configurations, potentially exceeding the resource-use ceiling of sole cropping systems (Wang et al., 2020; Zhang et al., 2025). Comparative analysis reveals that GM/S achieved its maximum LER (1.071) under the 4:4 high-density configuration, whereas FM/S reached its peak of 1.107 under the same row ratio but at medium density (**Figure 4**). Further analysis of partial land equivalent ratio (PLER) revealed no significant difference in the PLER of the main crops (grain maize and fresh maize) between GM/S and FM/S systems. However, the PLER of soybean intercropped with fresh maize was markedly higher than that of soybean intercropped with grain maize, and this difference may partly explain the higher overall LER of FM/S. This observation implies relatively milder interspecific competition and stronger complementarity in FM/S, which could allow the system to retain the productivity advantage of the main crop while alleviating competitive suppression on the subordinate crop.

NE quantifies the absolute yield advantage of intercropping systems over sole cropping (Zhang et al., 2021). In this study, the three-year mean NE of both intercropping systems was greater than zero (**Figure 7**), indicating a stable net yield advantage of intercropping over sole cropping at the system level. Specifically, GM/S achieved its maximum NE under the 4:4 high-density configuration, while FM/S did so under the 4:4 medium-density configuration (**Figure 7**), which closely coincided with their respective LER-optimal configurations (**Figure 4**). This observation points to a possible association between efficient resource use and net yield gain. The 4:4 row ratio may favor optimal canopy architecture and maximizes the border row effect, reducing competitive suppression while enhancing spatial–temporal niche differentiation, and could improve overall functional stability of the system.

**Figure 7:**
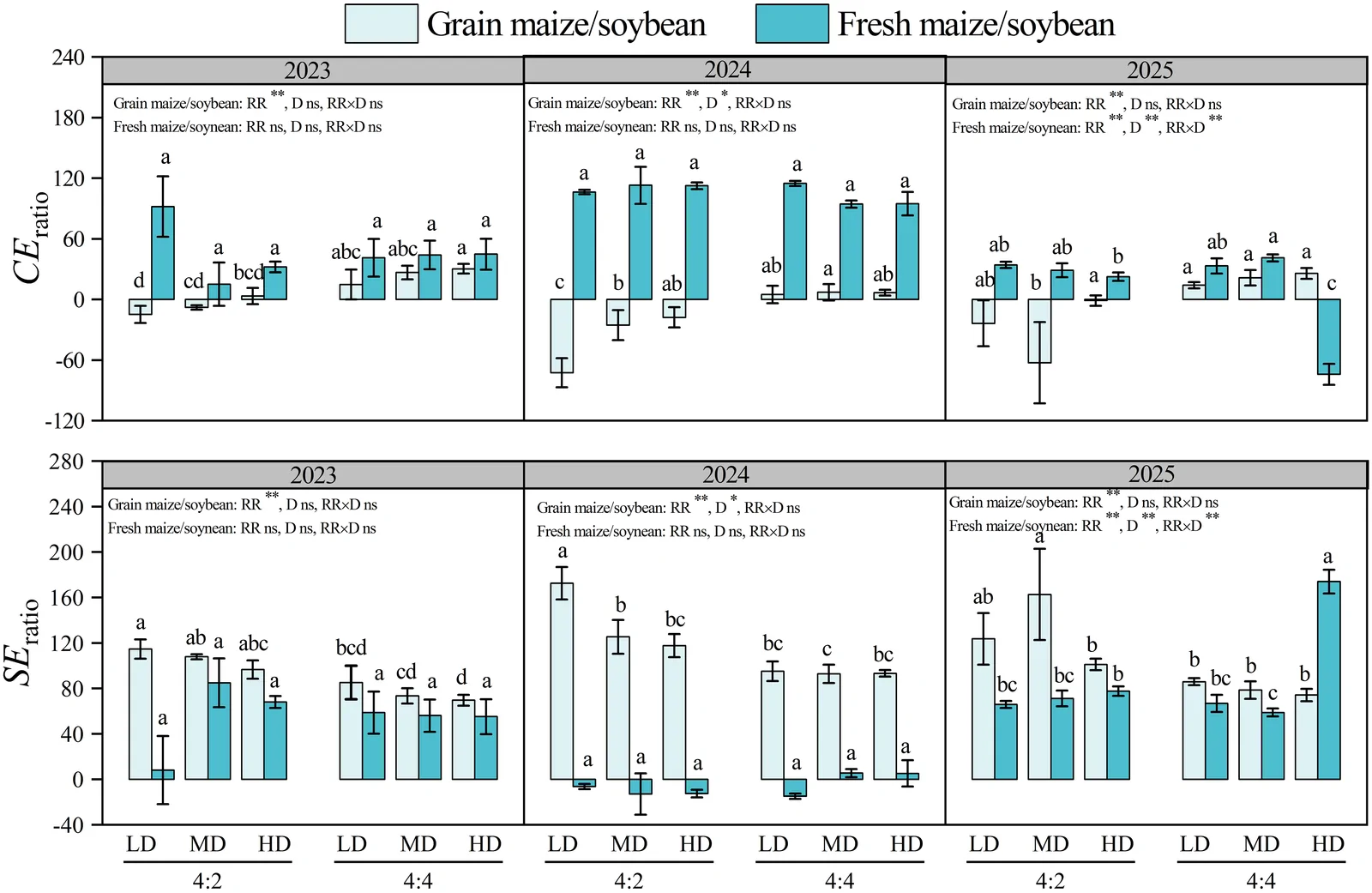
Contribution proportions of selection effect and complementarity effect in grain maize (or fresh maize)/soybean intercropping systems (2023–2025). Values are mean ± SE (n=3). * and ** indicate P < 0.05 and P < 0.01, respectively; ns, no significant difference. Different lowercase letters indicate significant differences at P < 0.05.

### 4.2 Interspecific competition: synergistic analysis of competitiveness, SE and CE

The balance of interspecific competition is a key factor determining whether the productivity advantage of intercropping systems can be stably achieved (Wu et al., 2023b; Ma et al., 2024). In this study, the CR of the main crops (grain maize/fresh maize) relative to soybean was greater than 1, and the A was greater than 0 in both intercropping systems (**Figure 5**), indicating that the main crops consistently dominate the interspecific competition with soybean, which may correspond to superior capacity to acquire and utilize limiting resources (including light, heat, water, and nutrients) (Wu et al., 2023a). Competitive intensity analysis revealed that grain maize in the GM/S system exerted strong competitive pressure on soybean, with a mean CR of 2.07 and a mean A of 0.62 (**Figure 5**). This was because the productivity advantage of GM/S was dominated by the SE, which contributed on average 98.12% of the NE over the three years (**Figures 6**, **7**). The system yield advantage relied almost entirely on the SE of grain maize, whereas the CE contributed minimally, peaking at only 22.04% (**Figures 6**, **7**). During the co-growth period, grain maize’s taller stature conferred superior light interception and greater root system competitiveness for soil resources, which may contribute to chronic suppression of soybean growth and failure to activate CE. Consequently, soybean suffered an average yield penalty of 37.32% relative to sole cropping (**Figure 3**). Supporting this interpretation, prior research demonstrated that persistent shading by tall crops significantly reduced photosynthetic efficiency and carbon assimilation in understory species (Lu et al., 2023); this mechanism may partly explain the severe yield depression observed in soybean under GM/S. In contrast, the competitiveness of fresh maize against soybean in the FM/S system was relatively moderate, with an average CR of 1.43 and an average A of 0.34 (**Figure 5**). This mild competitiveness reflects a balanced synergy between SE and CE, with their average contributions to NE being 54.68% and 45.32%, respectively (**Figures 6**, **7**). This balanced and coordinated pattern arises from the synergistic interplay between spatial resource partitioning and temporal niche differentiation. Spatially, optimized row ratio and density configurations confer a competitive advantage to fresh maize without unduly restricting soybean canopy development or root zone expansion, thereby preserving soybean growth capacity and allowing the selection effect to function positively. Temporally, the early harvest of fresh maize reduces interspecific competition during soybean late-stage growth. Based on three-year field observations, there is a subsequent growth phase of more than 20 days from fresh-maize harvest to soybean harvest, during which soybean can complete its seed-filling process free from fresh maize competition. As a result, soybean growth conditions in late season can be improved, which may help enhance carbon assimilation efficiency and photoassimilate allocation to seeds, evidenced by increased 100-seed weight and reduced shriveled grain rate (Vogel et al., 2021), reducing the average soybean yield penalty to 16.07% (**Figure 3**) and fully activating CE. The balanced development of the two effects effectively mitigates the negative impacts of interspecific competition and achieves coordinated coexistence between the main and subordinate crops, which may be one important factor why FM/S outperforms GM/S in NE and LER.

### 4.3 High productivity mechanism of FM/S systems: superiority of spatiotemporal dual regulation

The pronounced productivity advantage of FM/S over GM/S stems fundamentally from its dual-track regulation mechanism combining optimized spatial configuration and temporal niche differentiation, whereas GM/S relies solely on a single-track, space-only strategy centered on optimized row ratio and planting density configuration. Spatially, both systems improve resource use efficiency by optimizing population structure (Wang et al., 2021). GM/S achieves its optimal configuration under the 4:4 high-density treatment, while FM/S reaches its peak LER under the 4:4 medium-density treatment. Appropriate spatial configuration enables stratified and efficient capture and utilization of light energy, soil water, and nutrients through coordinated regulation of vertical canopy layering and root distribution (Li et al., 2020a; Homulle et al., 2022), fully demonstrating the population benefit derived from the synergistic optimization of row spacing and planting density (Yang et al., 2022). We hypothesize that the fresh maize in the FM/S system has a compact plant architecture and moderate stature, which may optimize light distribution within the intercropping canopy, reduces upper-canopy shading, and improves light availability for middle and lower strata. This reserves more photosynthetic resources for intercropped soybean, potentially resulting in a more pronounced spatial population optimization effect (Liu et al., 2017b). From a temporal perspective, the unique temporal niche differentiation in FM/S represents a core innovation distinguishing it from GM/S. Fresh maize has a markedly shorter growth period than grain maize, thereby reducing the duration of interspecific coexistence with soybean. Crucially, the early harvest of fresh maize creates a dedicated growth window during soybean’s critical seed-filling stage, triggering a robust compensatory growth response. This mechanism allows soybean to partially or fully offset earlier competitive suppression, leading to enhanced seed weight, thus increasing its PLER and consequently the overall LER (**Figure 4**). In contrast, soybean in GM/S coexists with grain maize throughout the entire growth period, remaining under continuous competitive suppression without a temporal release window. Consequently, soybean is unable to achieve recovery or compensatory growth, leading to sustained limitations in resource capture and use efficiency.

### 4.4 Optimal intercropping configuration: balancing productivity, economic benefits, and mechanization

Optimal intercropping configuration must balance productivity, economic benefits, and mechanization suitability—a key prerequisite for scaling intercropping technology from experimental plots to large-scale application (Wang et al., 2022; Al-Amin et al., 2024). Data averaged across the three-year study showed that both GM/S and FM/S achieved the highest economic benefits under the 4:2 high-density configuration (**Table 2**); however, their corresponding LER values were only 0.994 and 1.029, respectively—both near or below one, indicating suboptimal resource use and limited yield advantage over sole cropping. In contrast, LER peaked at 1.071 (GM/S, 4:4 high density) and 1.107 (FM/S, 4:4 medium density) (**Figure 4**), where resource use efficiency was highest and intercropping advantage was strongest. Notably, compared with their respective maximum economic benefits, the economic benefits under these two LER-optimal configurations were only 0.75% and 3.76% lower, respectively, with no significant differences. This indicates that both systems can achieve near-optimal economic benefits while ensuring high resource use efficiency under the 4:4 high-density (for GM/S) and 4:4 medium-density (for FM/S) configurations, respectively. From the perspective of practical production and mechanized operations, the 4:4 row ratio configuration features uniform maize and soybean row widths, balanced canopy structure, and superior field ventilation, making it highly compatible with standard widths of sowing, fertilizing, and harvesting machinery (Rajula Shanthy and Muthusamy, 2012). In contrast, the 4:2 configuration has too few soybean rows to match current agricultural machinery parameters, significantly increasing the difficulty of mechanized operations (including skipped passes, uneven application, or manual intervention) and substantially reducing its feasibility for large-scale mechanized farming. In conclusion, the 4:4 row ratio configuration combines higher resource use efficiency, good mechanization suitability, and acceptable economic benefits, better aligning with the goals of large-scale, labor-saving production. It is therefore recommended as the preferred configuration for both intercropping systems.

## 5 Conclusion

This study identifies the 4:4 medium-density configuration for FM/S as the optimal configuration: it achieves the highest LER of 1.107, near-maximal economic benefits, a manageable soybean yield reduction (16.07%), and full compatibility with mechanized field operations. For GM/S, the optimal configuration is the 4:4 high-density configuration; however, due to over-reliance on grain maize yield gain, the balance of interspecific competition is relatively poor. Compared with GM/S, FM/S exhibits superior performance in productivity, economic benefit, and ecological sustainability. Under their respective optimal configurations, FM/S outperforms GM/S by 3.36% in LER, 11.47% in NE, and 11.42% in economic benefit. The core advantage of FM/S stems from its dual-track regulation mechanism of “spatial configuration optimization + temporal niche differentiation”. This system is well suited for large-scale adoption in northern China and provides important theoretical support and practical technical references for optimizing cereal–legume intercropping systems. Derived from three-year observations at a single semi-arid rainfed site in northern China, these findings offer important theoretical support and practical technical references for optimizing cereal-legume intercropping systems, although broader multi-environment validation is needed prior to large-scale practical adoption.

## Data Availability

The original contributions presented in the study are included in the article/supplementary material. Further inquiries can be directed to the corresponding authors.
